# Supplementary material for: Heterogeneous receptor expression underlies non-uniform peptidergic modulation of olfaction in Drosophila
Source: Nat Commun. 2023 Aug 30;14:5280. doi: 10.1038/s41467-023-41012-3 (PMC10465596; doi:10.1038/s41467-023-41012-3)
Supplement: Supplementary file 1 — Supplementary Information [file 41467_2023_41012_MOESM1_ESM.pdf]

Supplementary Information for

Heterogeneous Receptor Expression Underlies Non-uniform Peptidergic Modulation of Olfaction in *Drosophila*

Tyler R. Sizemore[1,2*], Julius Jonaitis[1], and Andrew M. Dacks[1,3†]

[1]Department of Biology, Life Sciences Building, West Virginia University, Morgantown, WV, 26506, USA.
[2]Present Address: Department of Molecular, Cellular, and Developmental Biology, Yale Science Building, Yale University, New Haven, CT, 06520-8103, USA.
[3]Department of Neuroscience, West Virginia University, Morgantown, WV, 26506, USA.

Correspondence:
T.R.S (*email: tyler.sizemore@yale.edu) or
A.M.D. (†email: Andrew.Dacks@mail.wvu.edu)

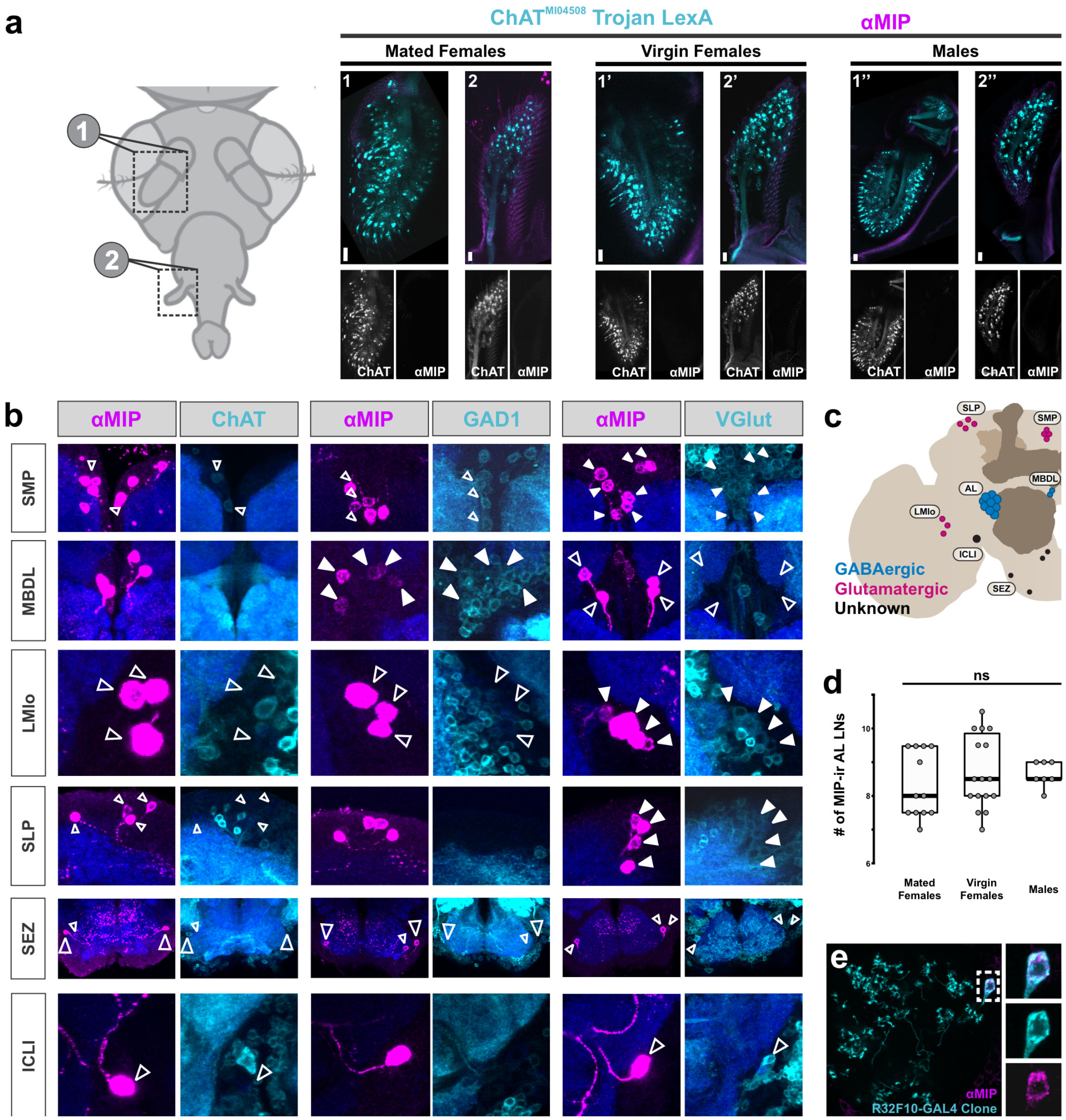

Supplementary Fig. 1 | Myoinhibitory peptide (MIP) colabeling with transgenic markers for GABAergic, cholinergic, and glutamatergic neurons in the *Drosophila* central brain.

(a) Regardless of sex or mating status, there are no MIP-immunoreactive (MIP-ir) OSNs in *Drosophila*. The left most diagram represents the imaging plane for all images to the right, wherein: **1-1”** = OSNs in the 3rd-antennal segment; **2-2”** = maxillary palp OSNs. High-contrast black-and-white images for each individual label (ChAT Trojan LexA-derived tdTomato or anti-MIP) are shown below each merged image (images in color). **(b)** MIPergic neurons in the antennal lobe (AL) **(Fig. 1)** and near the median bundle (MBDL) colabel with glutamic acid decarboxylase 1 (GAD1). MIPergic neurons in the superior medial and lateral protocerebrum (SMP and SLP, respectively) and near the lateral medial lobula (LMlo) colabel with vesicular glutamate transporter (VGlut). MIPergic neurons within the inferior contralateral

interneuron cluster (ICLI)[1] and SEZ do not colabel for ChAT, GAD1, or VGlut, and are likely tyraminergic (Tyr) based on scRNA-seq data[2].
(c) Schematic summarizing data from **(b)**, wherein several populations of MIP-immunoreactive neurons are also glutamatergic (MIP$^+$-VGlut$^+$ neurons in the SMP, LMlo, and SLP; magenta), two populations are also GABAergic (MIP$^+$-GAD1$^+$ neurons in the MBDL and AL) **(see also Fig. 1)**, and no MIP-immunoreactive neurons are cholinergic (colabel with ChAT). Except for the ICLI interneurons, soma locations are labeled according to the closest neuropil, or fascicle, according to established nomenclature[3]. **(d)** The number of MIP-ir AL LNs does not differ based on sex or mating status (p = 0.548, Kruskal-Wallis rank sum test). Cell counts: n = 12 brains, 24 ALs (mated female); n = 16 brains, 31 ALs (virgin female) ; n = 7 brains, 14 ALs (male). **(e)** Representative image of R32F10-GAL4 clone colabeled for MIP shows MIPergic LNs are bonafide patchy AL LNs. In all cases: neuropil was delineated by anti-DN-cadherin staining; open arrowheads = no colocalization; closed arrowheads = colocalization; scale bars = 10um.

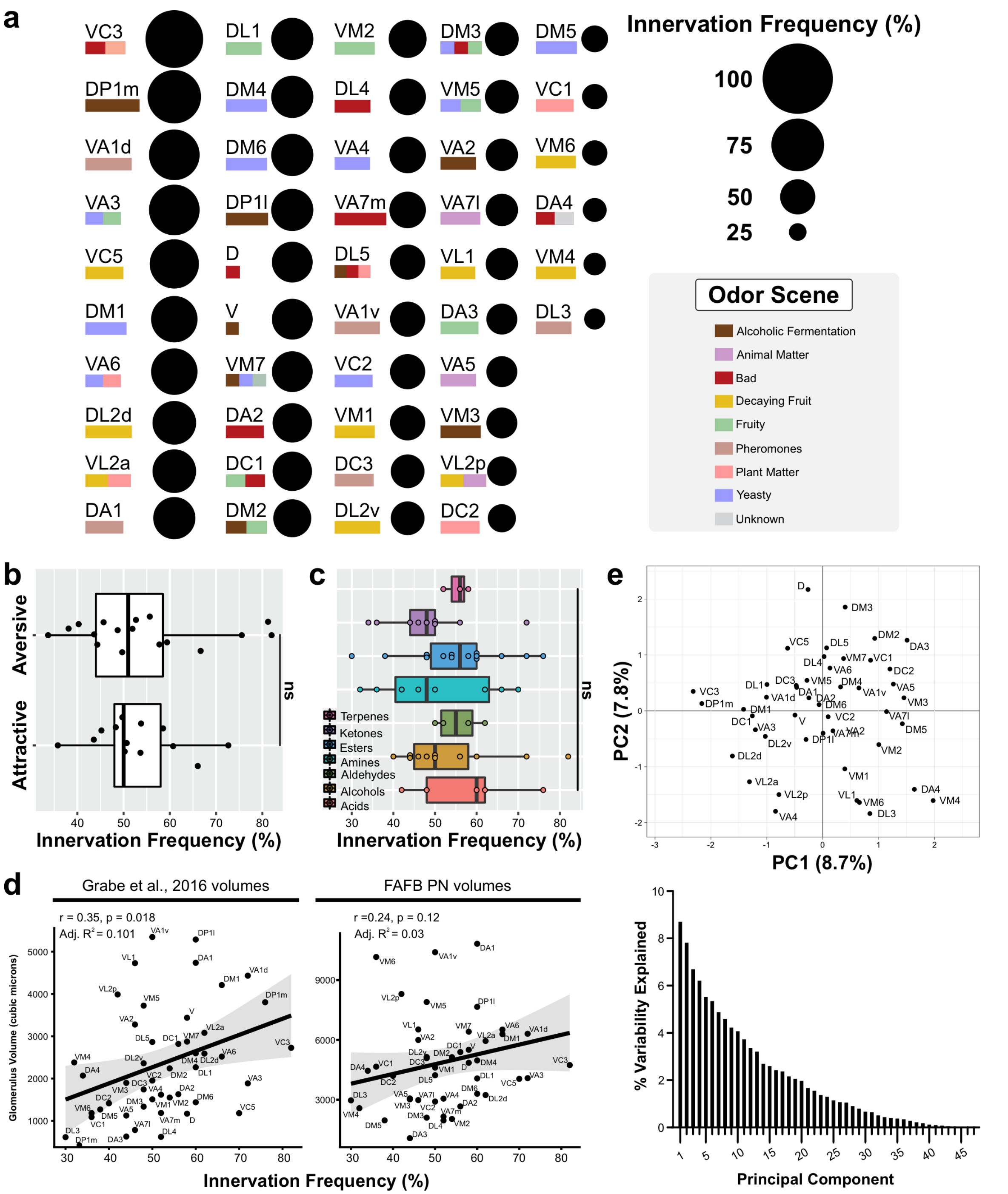

Supplementary Fig. 2 | MIPergic LNs do not preferentially innervate olfactory glomeruli based on odor-evoked behavioral valence, the odor-tuning properties of a given glomeruli's olfactory receptor neuron(s), or the size of the glomerulus.

(a) Dot plot representation of the frequency we find a given glomerulus is innervated by a single MIPergic LN clone. Rectangles underneath each glomerulus' name represents the "odor scene" of that glomerulus[4,5]. These are: alcoholic fermentation (brown); yeasty (blue); fruity (faded green); decaying fruit (yellow); plant matter (pink);

animal matter (pale purple); pheromones (chartreuse); dangerous (red); and, unknown (grey). **(b)** MIPergic LNs do not preferentially innervate glomeruli whose activity has been linked to attractive or aversive behavioral responses ($p = 0.991$, $n = 13$ ("attractive"), 16 ("aversive"), unpaired t-test with Welch's correction). **(c)** MIPergic LNs do not preferentially innervate glomeruli tuned to any particular odorant molecules ($p = 0.59$, Kruskal-Wallis rank sum test). Odorant molecule functional groups are color coded as follows: terpenes (magenta), ketones (purple), esters (blue), aromatics (aqua marine), amines (chartreuse), aldehydes (green), alcohols (brown), and acids (deep pink). **(d)** The frequency by which a MIPergic LN innervates a glomerulus is not correlated to the volume of the glomerulus (cubic microns). MIPergic LN innervation frequencies are significantly weakly correlated to glomerular volumes delineated by Grabe et al.[6] ($r = 0.35$, $p = 0.018$), but variations in MIPergic innervation frequencies across glomeruli do not correlate (adjusted $R^2 = 0.101$). Conversely, MIPergic LN innervation frequencies do not correlate with projection neuron-based glomerular volumes delineated from electron microscopy data[4,5] ($r = 0.24$, $p = 0.12$). **(e)** Principal components analysis (PCA) of MIPergic LN innervation patterns, where each data point represents MIPergic LN innervation patterns for each glomerulus. Bar graph represents the percentage of the variance explained by each principal component.

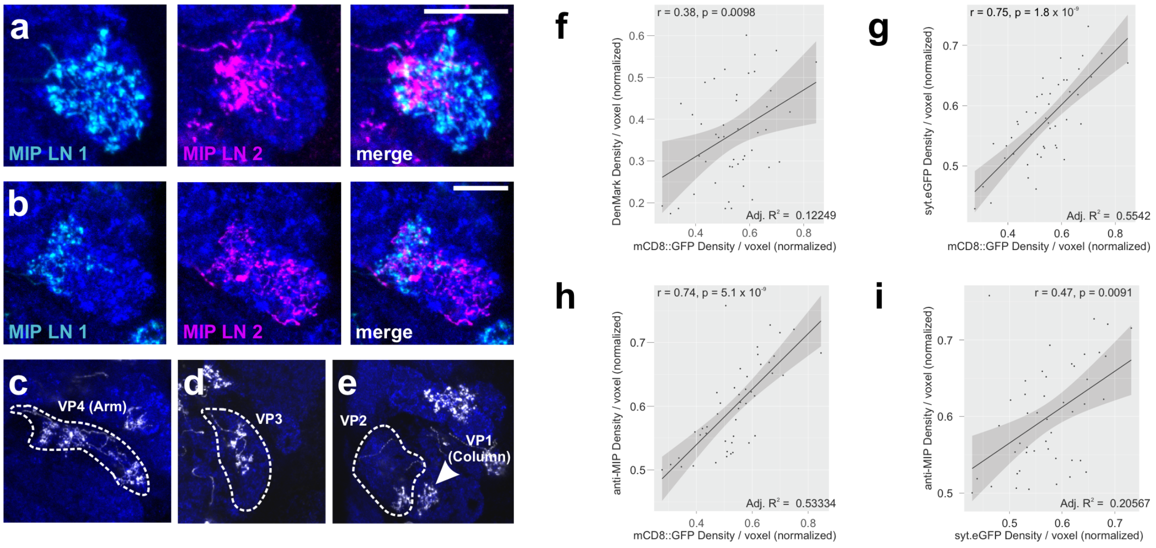

j

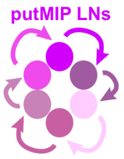

MIP LN 1	MIP LN 2	MIP LN 3	MIP LN 4	MIP LN 5	MIP LN 6	MIP LN 7	MIP LN 8	MIP LN 9	MIP LN 10	MIP LN 11	MIP LN 12	MIP LN 13	MIP LN 14
	0	89	12	14	0	83	0	12	57	0	97	8	4
1		113	12	7	0	83	0	12	47	0	125	9	0
13	9		13	16	4	0	9	7	0	2	1	3	4
10	11	25		0	9	35	13	2	4	9	12	0	14
20	13	17	0		9	5	8	0	19	4	23	1	9
0	1	55	9	11		126	1	20	164	1	90	6	2
2	5	0	18	9	9		3	11	0	3	0	6	4
1	0	55	13	8	0	66		11	69	0	109	6	1
17	8	12	0	0	10	23	6		19	0	19	0	18
4	6	0	5	19	12	0	6	9		2	0	7	1
1	0	46	18	5	0	31	1	1	71		81	9	5
5	2	0	15	14	4	0	15	8	0	9		6	4
7	7	4	1	0	8	6	1	0	13	5	8		5
3	3	75	18	4	0	84	0	25	86	0	67	5	

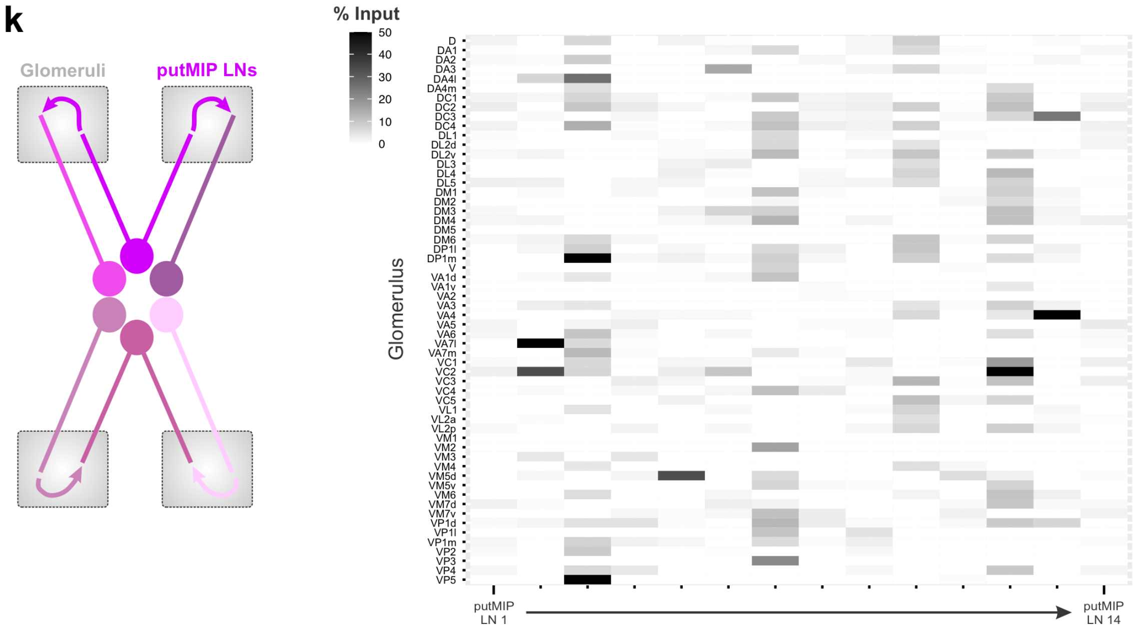

Supplementary Fig. 3 | Sister MIPergic LN and individual MIPergic LN connectivity dynamics.

(a & b) On average, ~12 glomeruli are co-innervated by sister MIPergic LN clones. In these examples, two distinct MIPergic LNs co-innervate DL2d and DP1l (respectively). For comparing sister MIPergic LN co-innervation patterns, n = 5 brains;

5 sister clones per brain. **(c-e)** Individual MIPergic LNs innervate thermo-/hygrosensory glomeruli. Branching from an individual MIPergic LN was observed invading the VP4 (formerly, “the arm”), VP3, VP2, and VP1 (formerly, “the column”). VP2-4 are designated by the hatched outline, while an arrowhead designates VP1. **(f-i)** DenMark, synaptotagmin-eGFP (syt.eGFP), and anti-myoinhibitory peptide immunoreactive puncta (anti-MIP) voxel density generally scale with MIPergic LN total cable voxel density within glomeruli. DenMark variations across glomeruli are significantly weakly correlated to the voxel density of total MIPergic LN cable within each glomerulus (r = 0.38, p = 0.0098). However, variations in DenMark voxel density across glomeruli do not correlate (adjusted R^2 = 0.12249). Synaptotagmin-eGFP and anti-MIP voxel density are significantly correlated with the voxel density of total MIPergic LN neurite volume (syt.eGFP: r = 0.75, p = $1.8x10^{-9}$; anti-MIP: r = 0.74, p = $5.1x10^{-9}$). The density of syt.eGFP and anti-MIP immunoreactive punctate are significantly correlated (p = 0.0091), but variations in either indicator across glomeruli are not (adjusted R^2 = 0.20567). In all cases, each data point represents the normalized mean indicator density within a given glomerulus and each line represents the linear regression model. **(j)** The number of synapses from one putMIP LN to all other putMIP LNs. **(k)** The amount of putative MIPergic LN reciprocal connectivity assessed within each glomerulus. Heatmap of the amount of input a given putMIP LN (x-axis) receives from all other putMIP LNs within every AL glomerulus as a function of the total amount of input that putMIP LN receives within a glomerulus. In all cases: neuropil was delineated by anti-DN-Cadherin staining; scale bars = 10um.

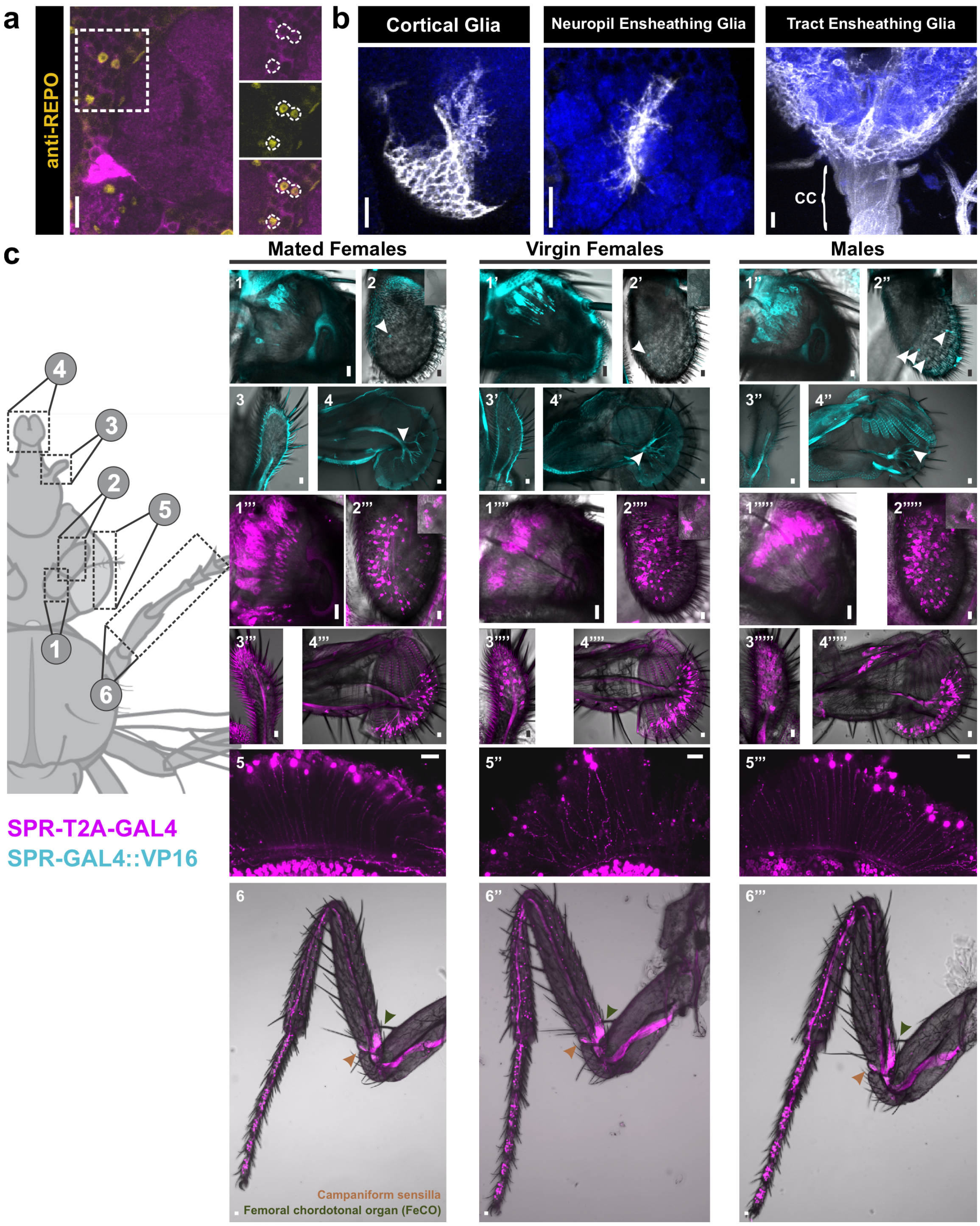

Supplementary Fig. 4 | SPR-T2A-GAL4 expression in several glia subtypes, and GAL4::VP16 and SPR-T2A-GAL4 expression throughout all primary sensory neurons.

(a) SPR-T2A-GAL4 colocalizes with the glial marker REPO (yellow). **(b)** SPR-T2A-GAL4 stochastic labeling reveals expression in cortical, neuropil ensheathing, and tract ensheathing glia. “CC” = Cervical Connective. **(c)** Expression patterns of the

bacterial artificial chromosome derived element SPR-GAL4::VP16 (cyan) and a CRISPR-Cas9 T2A-GAL4 insertion in the coding-intron of the sex peptide receptor (SPR-T2A-GAL4, magenta) in all sensory afferents in mated females, virgin females, and males. The left most diagram represents imaging plane for all images to the right, wherein: **1-1'''''** = auditory afferents; **2-2'''''** = olfactory, thermal, and hygrosensory afferents; **3-3'''''** = olfactory afferents; **4-4'''''** = gustatory afferents; **5-5'''** = visual afferents; **6-6'''** = proprioceptive and gustatory afferents. Driver expression in visual afferents and proprioceptive/gustatory afferents (in T1) were only tested for SPR-T2A-GAL4. Arrowhead(s) in **2-2'''** and **4-4'''** highlight the few neurons the express SPR-GAL4::VP16 in the 3rd-antennal segment (olfactory, thermal, and hygrosensory afferents), and the labellum (gustatory afferents), respectively. Neuron(s) that innervate the sacculus, a thermal/hygrosensory organ in the 3rd-antennal segment, are presented in the insets in the top right of **2-2'''''**.

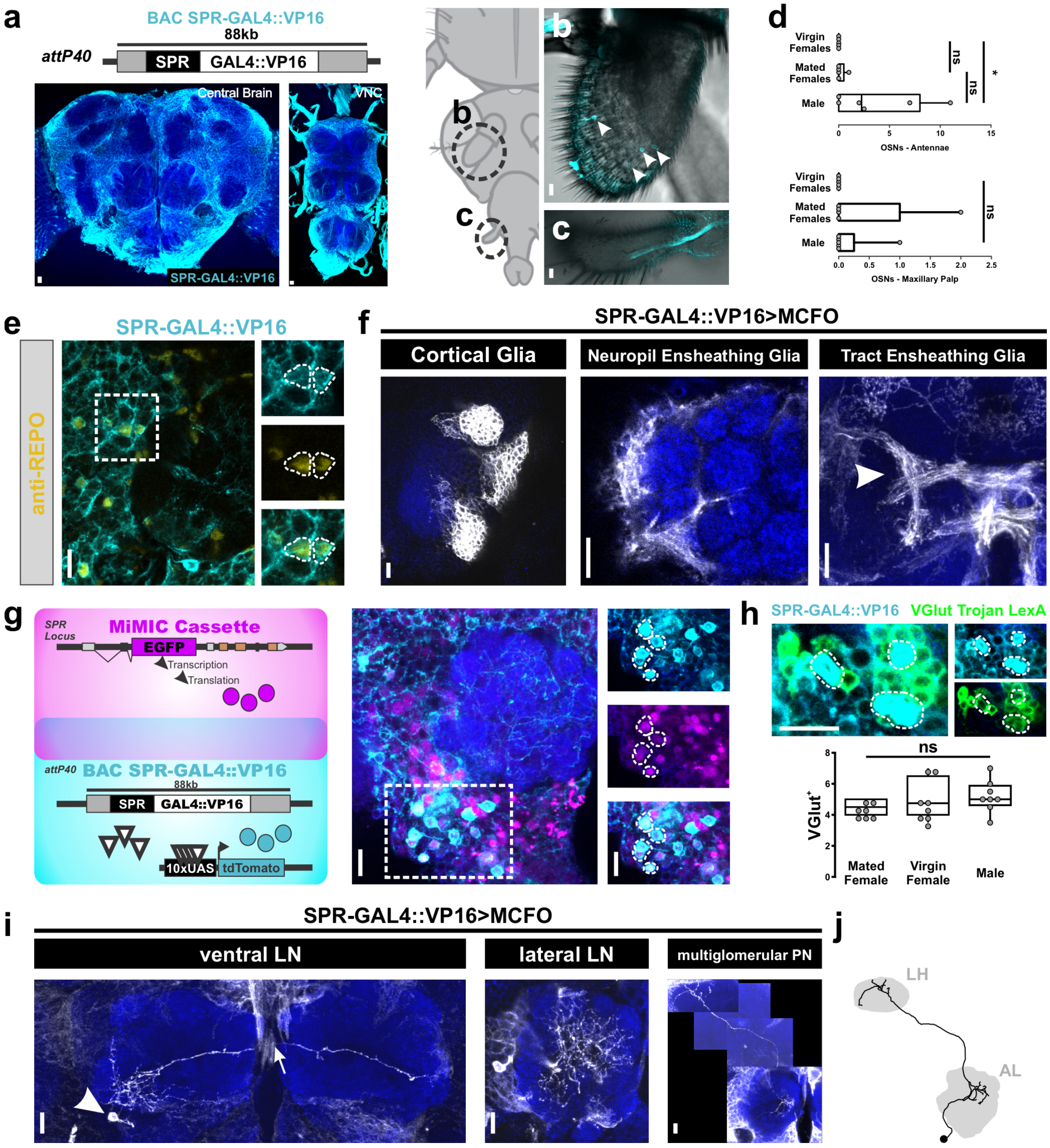

Supplementary Fig. 5 | SPR-GAL4::VP16 expression throughout central brain circuitry with emphasis on AL expression.

(a) Sex peptide receptor expression (SPR; cyan) as revealed using a bacterial artificial chromosome derived GAL4::VP16 element[7]. Note that this element contains the SPR locus and much of the surrounding genomic locus (~88kb total), and the GAL4::VP16 coding sequence was later inserted before the SPR stop site[7]. This element was then reintroduced at the attp40 landing site[7]. **(b-d)** SPR-GAL4::VP16 expression (cyan) in OSNs housed in the 3rd-antennal segment and maxillary palp. Female mating status does not affect the number of SPR-GAL4::VP16-positive cells in antennae (p = 0.63; Holm-adjusted Dunn test), but males have significantly more SPR-GAL4::VP16-positive cells in their antennae than virgin females (p = 0.05; Holm-

adjusted Dunn test). However, the number of SPR-GAL4::VP16-positive cells in the maxillary palp does not differ based on sex or mating status ($p = 0.59$; Kruskal-Wallis rank sum test). The discrepancy in the number of neurons of a given type observed between the SPR-T2A-GAL4 **(see Fig. 7c & 7d)** versus the SPR-GAL4::VP16 drivers is likely a result of the non-native chromosomal topology, as well as potentially missing enhancer/repressor elements, of the SPR-GAL4::VP16 driver. **(e)** SPR-GAL4::VP16 (cyan) colocalizes with the general glial marker *reverse polarity* (anti-REPO; yellow). **(f)** SPR-GAL4::VP16 stochastic labeling highlights several glial subtypes, including cortical, neuropil ensheathing, and tract ensheathing glia. **(g)** Several ventral AL neurons are labeled through intersectional genetics experiments between an EGFP-insertion in the endogenous non-coding intron of SPR (MiMIC Cassette; magenta) and SPR-GAL4::VP16 (cyan). **(h)** At least a portion of the ventral AL neurons labeled by SPR-GAL4::VP16 are ventral glutamatergic LNs. The number of vesicular glutamate transporter-positive ($VGlut^+$) SPR-GAL4::VP16 neurons does not statistically differ based on sex or mating status ($p = 0.28$, $n = 8$ (virgin females), 7 (mated females), and 8 (males); Kruskal-Wallis rank sum test). **(i)** SPR-GAL4::VP16 stochastic labeling confirms expression in ventral LNs (arrowhead = soma, contralateral projection = arrow), at least one lateral LN, and at least one ventral multiglomerular PN could be resolved. **(j)** Skeleton representation of the aforementioned SPR-GAL4::VP16 multiglomerular PN. In all cases: neuropil was delineated with anti-DN-cadherin staining; scale bars = 10um.

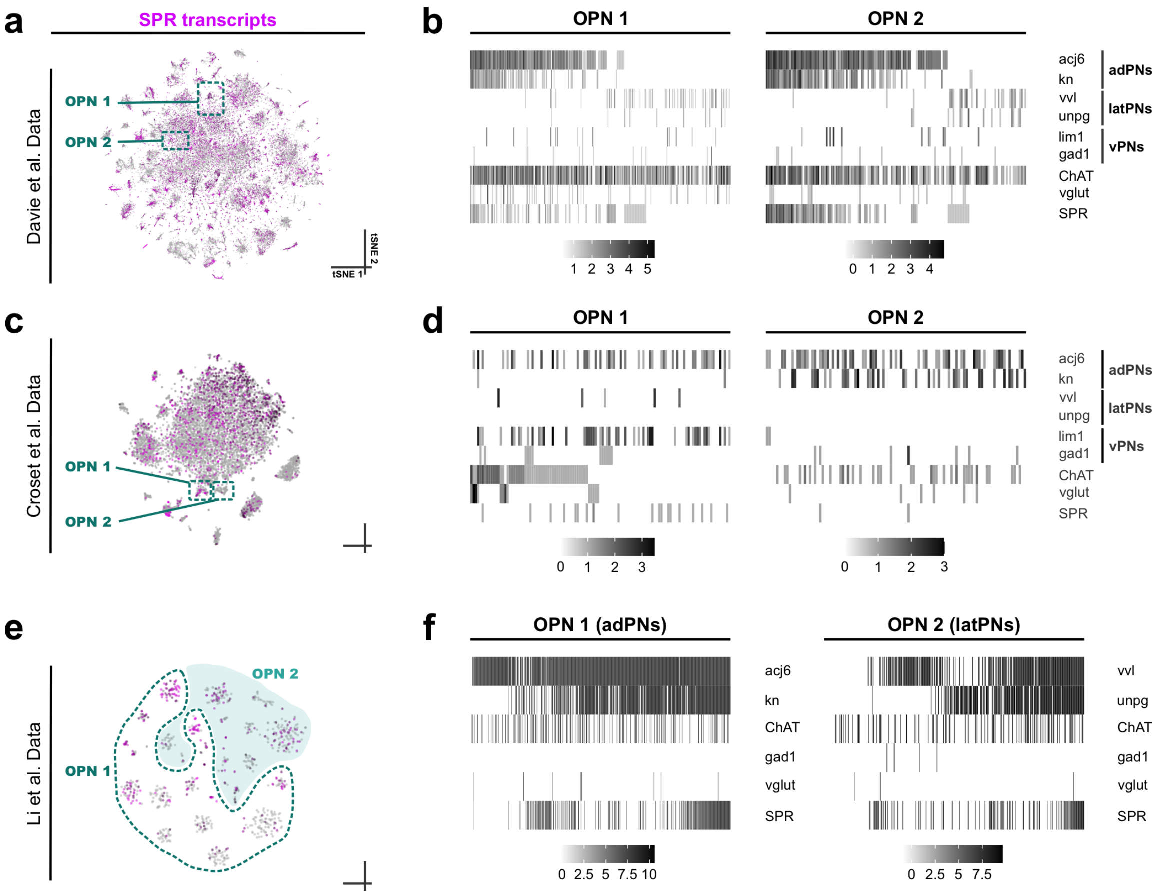

Supplementary Fig. 6 | Sex peptide receptor (SPR) expression in independently generated projection neuron single-cell RNA-sequencing (scRNA-seq) datasets. **(a)** T-distributed stochastic neighbor embedding (tSNE) plot showing SPR expression (log-transformed and counts per million (CPM) normalized), wherein higher transcript levels are deeper magenta. **(b)** Heatmap showing transcript levels of anterodorsal projection neuron (adPN) marker genes (*acj6* and *kn*), lateral projection neuron (latPN) marker genes (*vvl* and *unpg*), ventral projection neuron (vPN) marker genes (*lim1* and *gad1*), choline acetyltransferase (ChAT), vesicular glutamate transporter (*vglut*), and SPR in olfactory projection neuron (OPN) clusters previously identified[8]. **(c)** As in **a**, visualization of SPR expression in Croset et al.[2] scRNA-seq dataset. **(d)** As in **b**, heatmap showing transcript levels of various genes in OPN clusters previously identified[2]. **(e)** As in **a**, visualization of SPR expression in Li et al.[9] OPN scRNA-seq data. **(f)** As in **b**, heatmap representation of transcript levels within adPN (OPN 1) and latPN (OPN 2) scRNA-seq clusters. Cluster boundaries were previously identified[9]. In all cases: Transcript levels are CPM normalized and log-transformed.

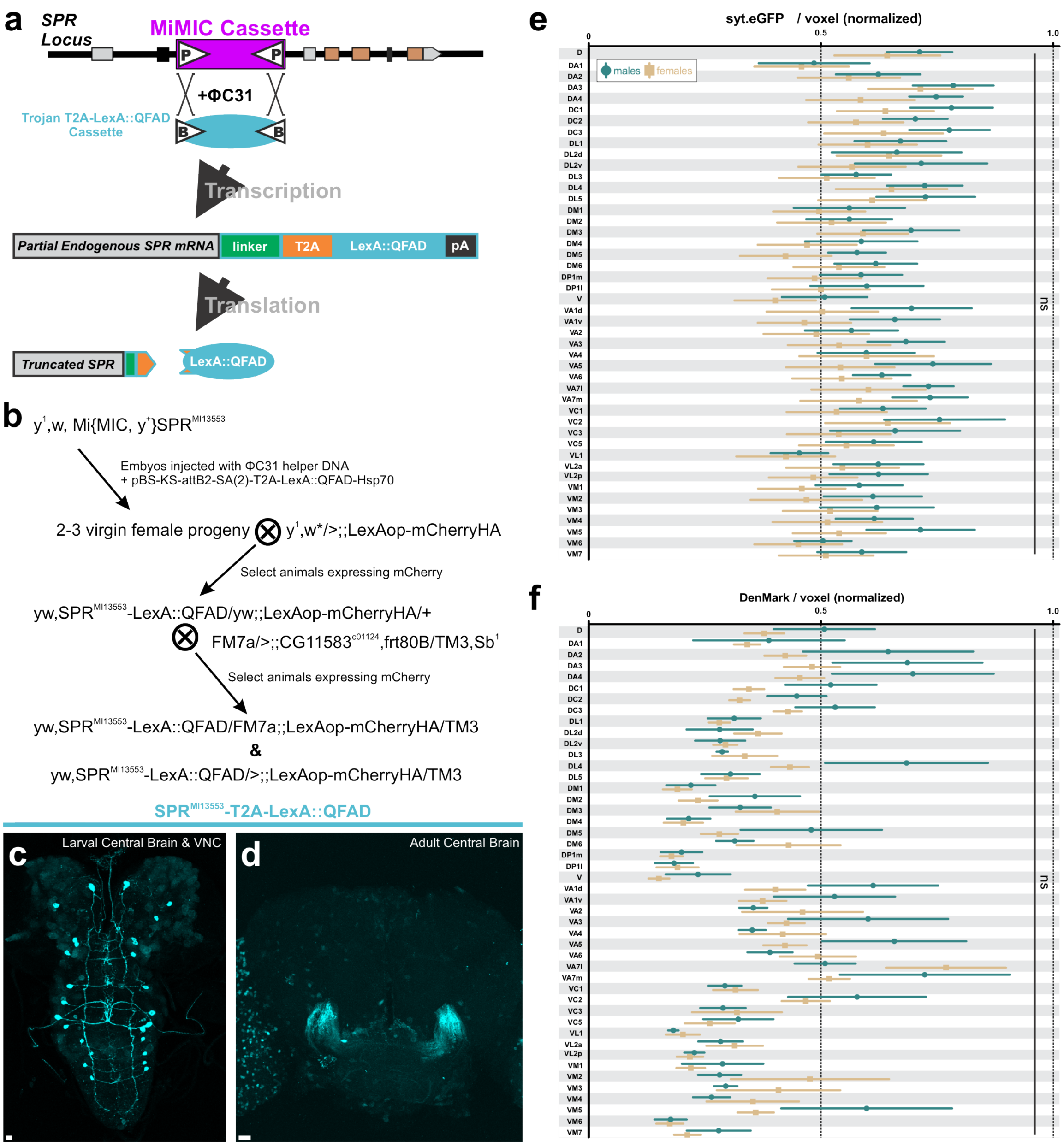

Supplementary Fig. 7 | Generation of a sex peptide receptor (SPR) LexA::QFAD driver line via dual microinjection of a Trojan exon construct and phiC31 recombinase.

(a) Schematic representation of MiMIC cassette exchange for LexA::QFAD Trojan exon cassette, and subsequent LexA::QFAD expression in all cells that produce the sex peptide receptor (SPR). **(b)** Crossing scheme used to establish SPR^{MI13553}-T2A-LexA::QFAD transgenics. **(c)** SPR^{MI13553}-T2A-LexA::QFAD expression (cyan) in the larval central brain and ventral nerve cord. **(d)** SPR^{MI13553}-T2A-LexA::QFAD expression (cyan) in the adult central brain. Note that while soma labeled by this driver are faintly reliably resolvable, neural processes are generally unresolvable in the adult. **(e & f)** R32F10-GAL4 synaptotagmin-eGFP (syt.eGFP) and DenMark voxel density do not display sexual dimorphism across glomeruli (syt.eGFP: $p = 0.0634$;

DenMark: p = 0.4347; n = 3 brains, 6 ALs (male), 4 brains, 8 ALs (females); two-way ANOVA). In all cases, scale bars = 10um.

Supplementary References

1. Jiang, H. *et al.* Natalisin, a tachykinin-like signaling system, regulates sexual activity and fecundity in insects. *Proc Natl Acad Sci U S A* **110**, (2013).
2. Croset, V., Treiber, C. D. & Waddell, S. Cellular diversity in the Drosophila midbrain revealed by single-cell transcriptomics. *Elife* **7**, 1–31 (2018).
3. Ito, K. *et al.* A systematic nomenclature for the insect brain. *Neuron* **81**, 755–765 (2014).
4. Bates, A. S. *et al.* Complete Connectomic Reconstruction of Olfactory Projection Neurons in the Fly Brain. *Current Biology* **30**, 3183-3199.e6 (2020).
5. Schlegel, P. *et al.* Information flow, cell types and stereotypy in a full olfactory connectome. *Elife* (2021) doi:10.1101/2020.12.15.401257.
6. Grabe, V. *et al.* Elucidating the Neuronal Architecture of Olfactory Glomeruli in the Drosophila Antennal Lobe. *Cell Rep* **16**, 3401–3413 (2016).
7. Ameku, T. *et al.* Midgut-derived neuropeptide F controls germline stem cell proliferation in a mating-dependent manner. *PLoS Biol* **16**, e2005004 (2018).
8. Davie, K. *et al.* A Single-Cell Transcriptome Atlas of the Aging Drosophila Brain. *Cell* 982–998 (2018) doi:10.1016/j.cell.2018.05.057.
9. Li, H. *et al.* Classifying Drosophila Olfactory Projection Neuron Resource Classifying Drosophila Olfactory Projection Neuron Subtypes by Single-Cell RNA Sequencing. *Cell* **171**, 1206-1207.e22 (2017).
