# Supplementary material for: Heterogeneous receptor expression underlies non-uniform peptidergic modulation of olfaction in Drosophila
Source: Nat Commun. 2023 Aug 30;14:5280. doi: 10.1038/s41467-023-41012-3 (PMC10465596; doi:10.1038/s41467-023-41012-3)
Supplement: Supplementary file 3 — Description of Additional Supplementary Files [file 41467_2023_41012_MOESM3_ESM.pdf]

Description of Additional Supplementary Files

File Name: Supplementary Data 1
Description: Sheet 1 (Genotypes by Figure). List of the *Drosophila melanogaster* genotypes for each figure. Sheet 2 (SPR-T2A-GAL4$^+$ AL Neurons). List summarizing SPR-T2A-GAL4 clonal analysis results (see Fig. 7). For each identified antennal lobe glomerulus, filled in cells indicate which principle neuron class a sparsely-labeled SPR-T2A-GAL4 expressing neuron belongs to. OSN(s): olfactory sensory neuron(s); PN(s): projection neuron(s); LN(s): local interneuron(s).
