# Supplementary material for: Heterogeneous receptor expression underlies non-uniform peptidergic modulation of olfaction in Drosophila
Source: Nat Commun. 2023 Aug 30;14:5280. doi: 10.1038/s41467-023-41012-3 (PMC10465596; doi:10.1038/s41467-023-41012-3)
Supplement: Supplementary file 5 — Reporting Summary [file 41467_2023_41012_MOESM5_ESM.pdf]

nature portfolio

Corresponding author(s): Tyler Sizemore
Andrew Dacks

Last updated by author(s): Aug 10, 2023

# Reporting Summary

Nature Portfolio wishes to improve the reproducibility of the work that we publish. This form provides structure for consistency and transparency in reporting. For further information on Nature Portfolio policies, see our Editorial Policies and the Editorial Policy Checklist.

## Statistics

For all statistical analyses, confirm that the following items are present in the figure legend, table legend, main text, or Methods section.

| n/a | Confirmed | |
|---|---|---|
| ☐ | ☒ | The exact sample size (*n*) for each experimental group/condition, given as a discrete number and unit of measurement |
| ☐ | ☒ | A statement on whether measurements were taken from distinct samples or whether the same sample was measured repeatedly |
| ☐ | ☒ | The statistical test(s) used AND whether they are one- or two-sided<br>*Only common tests should be described solely by name; describe more complex techniques in the Methods section.* |
| ☐ | ☒ | A description of all covariates tested |
| ☐ | ☒ | A description of any assumptions or corrections, such as tests of normality and adjustment for multiple comparisons |
| ☐ | ☒ | A full description of the statistical parameters including central tendency (e.g. means) or other basic estimates (e.g. regression coefficient) AND variation (e.g. standard deviation) or associated estimates of uncertainty (e.g. confidence intervals) |
| ☐ | ☒ | For null hypothesis testing, the test statistic (e.g. *F*, *t*, *r*) with confidence intervals, effect sizes, degrees of freedom and *P* value noted<br>*Give P values as exact values whenever suitable.* |
| ☒ | ☐ | For Bayesian analysis, information on the choice of priors and Markov chain Monte Carlo settings |
| ☐ | ☒ | For hierarchical and complex designs, identification of the appropriate level for tests and full reporting of outcomes |
| ☐ | ☒ | Estimates of effect sizes (e.g. Cohen's *d*, Pearson's *r*), indicating how they were calculated |

*Our web collection on statistics for biologists contains articles on many of the points above.*

## Software and code

Policy information about availability of computer code

| | |
|---|---|
| Data collection | Light microscopy data were collected with an Olympus BX61 confocal microscope running Fluoview FV1000 software with either a 40x UPlanFL-N or 60x PlanApo-N oil-immersion objective, or a 40x/1.25 UPlanSApo Silicone-immersion oil objective.<br><br>Single photon and two photon live-imaging data were collected with cellSens Dimension (v.2) and ScanImage (v.5.5), respectively. |
| Data analysis | Connectomics analyses were performed using Python (v.3.9) & R (v.4.1.3). The relevant code is openly-available on GitHub for each coding language's respective packages. Moreover, highly-detailed vignettes & examples are available for each repository. These are: natverse (https://github.com/natverse); hemibrainr (https://github.com/natverse/hemibrainr); neuprintr (https://github.com/natverse/neuprintr); cloud-volume (https://github.com/seung-lab/cloud-volume); navis (https://github.com/navis-org/navis); and, neuprint-python (https://github.com/connectome-neuprint/neuprint-python).<br><br>Live-imaging analyses to establish the background subtracted ΔF/F signal of a given live-imaging trial was performed using MATLAB (2018-2021). The relevant code was graciously provided by Marco Gallio (Northwestern University) and has been previously established and described (see Frank et al. 2015; Frank et al. 2017; Alpert et al. 2020). ΔF/F values from many glomeruli, across several animals, were then concatenated using Microsoft Excel before being imported into R (v.4.1.3). Once imported into R, these data were used for calculating the area under the ΔF/F curve (AUC), peak response, testing the relevant statistical question(s), and then plotted.<br><br>Light microscopy data were generally analyzed using VAA3D (v.3.20) and Fluorender (v.2.26.3). MIPergic LN puncta voxel density measurements were performed using a previously established and described FIJI (v.2.0.0) Segmentation Editor extension script that was graciously provided by Rachel Wilson (Harvard) (see Hong and Wilson 2015). These voxel density values were then aggregated using Microsoft Excel before being imported into R for statistical testing and plotting. |

Any custom code of ours used to analyze and plot the data is openly-available on GitHub at: https://github.com/tsizemo2/Sizemoreetal2023.

For manuscripts utilizing custom algorithms or software that are central to the research but not yet described in published literature, software must be made available to editors and reviewers. We strongly encourage code deposition in a community repository (e.g. GitHub). See the Nature Portfolio guidelines for submitting code & software for further information.

## Data

Policy information about availability of data

All manuscripts must include a data availability statement. This statement should provide the following information, where applicable:

- Accession codes, unique identifiers, or web links for publicly available datasets
- A description of any restrictions on data availability
- For clinical datasets or third party data, please ensure that the statement adheres to our policy

All source data from R32F10-GAL4 stochastic labeling experiments, DenMark/syt.eGFP/MIP/mCD8-GFP voxel density, putative MIPergic LN hemibrain extended data, physiology measurements, as well as a summary of which AL principal neuron type(s) from which glomeruli express SPR-T2A-GAL4 are provided in the Source Data file.

Original confocal scans for all light microscopy images in the main and supplementary figures can be found at: https://zenodo.org/10.5281/zenodo.8127341.

The hemibrain electron microscopy is publicly available and can be viewed on neuPRINT at https://neuprint.janelia.org/ (hemibrain version 1.2.1). Single-cell RNA sequencing data are publicly available and can be viewed on SCope at https://scope.aertslab.org/.

## Human research participants

Policy information about studies involving human research participants and Sex and Gender in Research.

| | |
|---|---|
| Reporting on sex and gender | N/A |
| Population characteristics | N/A |
| Recruitment | N/A |
| Ethics oversight | N/A |

Note that full information on the approval of the study protocol must also be provided in the manuscript.

# Field-specific reporting

Please select the one below that is the best fit for your research. If you are not sure, read the appropriate sections before making your selection.

☒ Life sciences ☐ Behavioural & social sciences ☐ Ecological, evolutionary & environmental sciences

For a reference copy of the document with all sections, see nature.com/documents/nr-reporting-summary-flat.pdf

# Life sciences study design

All studies must disclose on these points even when the disclosure is negative.

**Sample size**

Generally, the number of flies to be used for experiments are not a limiting factor, therefore no statistical power analyses were used to predetermine sample sizes. Instead, sample sizes were chosen based on conventions in our field for standard sample sizes, as well as published precedent for similar experiments. Sample sizes for most light microscopy data were based on Sizemore and Dacks 2016, Coates et al. 2020, and Hong and Wilson 2015. To effectively capture all individual R32F10-GAL4 AL LNs at least once during stochastic labeling experiments, the sample size was chosen based on the statistical probability theorem called, "the coupon collector problem" (Erdős and Rényi 1961).

The number of AL LNs from the hemibrain electron microscopy dataset analyzed were chosen based on which neurons were: (1) AL LNs; (2) previously shown to belong to the patchy AL LN subtype (Schlegel et al. 2020); (3) receive direct input from the contralaterally, projecting, serotonin-immunoreactive deutocerebral neurons (CSDns), as all MIPergic LNs express the 5-HT1A serotonin receptor (Sizemore and Dacks 2016) and form connections with the CSDns (Coates et al 2017); (4) had a morphological similarity score of >0.80 for AL LNs labeled by R32F10-GAL4; and, (5) were considered "Traced," the hemibrain's highest level of tracing completeness and confidence.

Sample sizes for live-imaging physiology data were based on Ignell et al. 2009, Frank et al. 2017, Ko et al. 2015, Oh et al. 2014, Badel et al. 2016, Diaz et al. 2019, Zandawala et al. 2021, and Lin et al. 2022.

**Data exclusions**

No data were excluded from analysis.

| | |
|---|---|
| Replication | Where possible, multiple complementary methods were used to independently test a given hypothesis in parallel. All experimental repeats were biological replicates (e.g., different fly brains were imaged in live-imaging physiology), and were performed over the course of several days to confirm reproducibility.<br><br>For light microscopy experiments, the number of flies of the appropriate genotype used varied from 4-41 depending on the experiment being performed. To assess questions pertaining to the colocalization/co-expression of two or more indicators, 4-23 flies of the appropriate genotype were used. To assess the number of SPR-T2A-GAL4 expressing olfactory afferents, we used 17-18 flies. We used 4-16 flies of the appropriate genotype and appropriate sex/mating status when assessing sexual dimorphism and/or mating status-dependent changes in MIP expression in primary olfactory neurons, the number of MIPergic AL LNs, SPR driver expression in sensory afferents, and MIPergic LN DenMark/syt.eGFP puncta density. For R32F10-GAL4 stochastic labeling experiments, 41 flies were used. All attempts at replication were successful.<br><br>For live-imaging physiology experiments, 12-40 odor presentation trials from 3-10 animals of the appropriate genotype were used. All attempts at replication were successful. |
| Randomization | The order of odors tested during MIPergic LN odor panel responses was randomly chosen. Flies were group-housed based on genotype, and individual animals of the given genotype were randomly selected from these groups. |
| Blinding | The experimenter was not blind to the animal's genotype in this report. In order to test/target/record from specific neurons, different effectors were genetically misexpressed in targeted cells. In doing so, the animal's genotype would be obvious to the experimenter based on the pattern of labeling/fluorescence/etc. Genotype blinding was unnecessary for live-imaging analyses; the same automated code was equally applied across all animals, regardless of the animal's genotype. |

# Reporting for specific materials, systems and methods

We require information from authors about some types of materials, experimental systems and methods used in many studies. Here, indicate whether each material, system or method listed is relevant to your study. If you are not sure if a list item applies to your research, read the appropriate section before selecting a response.

## Materials & experimental systems

| n/a | Involved in the study |
|---|---|
| ☐ | ☒ Antibodies |
| ☒ | ☐ Eukaryotic cell lines |
| ☒ | ☐ Palaeontology and archaeology |
| ☐ | ☒ Animals and other organisms |
| ☒ | ☐ Clinical data |
| ☒ | ☐ Dual use research of concern |

## Methods

| n/a | Involved in the study |
|---|---|
| ☒ | ☐ ChIP-seq |
| ☒ | ☐ Flow cytometry |
| ☒ | ☐ MRI-based neuroimaging |

## Antibodies

| | |
|---|---|
| Antibodies used | Table 1 summarizes the source/manufacturer, catalog number, and RRID (if relevant) for every antibody used in this study. These are:<br>Rabbit anti-RFP (Rockland, 600-401-379; RRID: AB_2209751)<br>Rabbit anti-DsRed (Clontech, 632496; RRID: AB_10013483)<br>Rat anti-DN-Cadherin (DSHB, DN-Ex #8; RRID: AB_528121)<br>Rabbit anti-GFP (Thermo Fisher Scientific, A-11122; RRID: AB_ 221569)<br>Chicken anti-GFP (Abcam, ab13970; RRID: AB_300798)<br>Rabbit anti-Hemagglutinin (Cell Signaling Technology, 3724; RRID: AB_1549585)<br>Mouse anti-V5-Tag::DyLight550 (BioRad, MCA1360D550GA; RRID: AB_2687576)<br>Rat anti-FLAG (Novus Bio, NBP1-06712SS; RRID: AB_1625982)<br>Mouse anti-Bruchpilot (DSHB, nc82; RRID: AB_2314866)<br>Rabbit anti-Myoinhibitory Peptide (MIP) (Manfred Eckert - gift from Christian Wegener; RRID: AB_2314803)<br>Rat anti-Embryonic lethal abnormal vision (ELAV) (DSHB; RRID: AB_528218)<br>Mouse anti-Reversed polarity (REPO) (DSHB; RRID: AB_528448)<br>Goat anti-Rabbit AlexaFluor 488 (Thermo Fisher Scientific, A-11008; RRID: AB_ 143165)<br>Donkey anti-Chicken AlexaFluor 488 (Jackson ImmunoResearch Laboratories, Inc., 703-545-155; RRID: AB_2340375)<br>Donkey anti-Rabbit AlexaFluor 546 (Thermo Fisher Scientific, A-10040; RRID: AB_2534016)<br>Goat anti-Mouse AlexaFluor 546 (Thermo Fisher Scientific, A-11030; RRID: AB_2534089)<br>Goat anti-Rabbit AlexaFluor 633 (Thermo Fisher Scientific, A-21070; RRID: AB_2535731)<br>Goat anti-Mouse AlexaFluor 633 (Thermo Fisher Scientific, A-21050; RRID: AB_2535718)<br>Donkey anti-Rat AlexaFluor 647 (Abcam, ab150155) |
| Validation | Rabbit anti-RFP: This commercial antibody was verified by immunoelectrophoresis to ensure the antibody binds to the antigen stated. https://www.rockland.com/categories/primary-antibodies/rfp-antibody-pre-adsorbed-600-401-379/<br>Rabbit anti-DsRed: The quality and performance of this commercial antibody was tested by Western blot analysis. https://www.takarabio.com/products/antibodies-and-elisa/fluorescent-protein-antibodies/red-fluorescent-protein-antibodies?catalog=632496<br>Rabbit anti-GFP: This commercial antibody was verified by relative expression to ensure the antibody binds to the antigen stated. https://www.thermofisher.com/antibody/product/GFP-Antibody-Polyclonal/A-11122 |

Rabbit anti-Hemagglutinin: This commercial antibody was verified by SimpleChIP Enzymatic Chromatin IP Kits to bind to the antigen stated. https://www.cellsignal.com/products/primary-antibodies/ha-tag-c29f4-rabbit-mab/3724
Mouse anti-V5-Tag::DyLight550: Mouse anti V5-Tag, clone SV5-Pk1 recognizes the sequence, IPNPLLGLD, present on the P/V proteins of the paramyxovirus, SV5 (Dunn et al.1999). Clone SV5-Pk1 is used to detect recombinant proteins, some of which include transmembrane and secreted proteins, that have labeled with tags containing this sequence (Randall et al.1993 and Zhao et al. 2005). https://www.bio-rad-antibodies.com/monoclonal/viral-v5-tag-antibody-sv5-pk1-mca1360.html?f=purified
Rat anti-FLAG: This commercial antibody was verified by Western blot analysis and immunohistochemistry to ensure the antibody binds to the antigen stated. https://www.novusbio.com/products/dykddddk-epitope-tag-antibody-l5_nbp1-06712
Mouse anti-Bruchpilot: This antibody was developed by Wagh et al., 2006 (Neuron) and is distributed by DSHB. https://dshb.biology.uiowa.edu/nc82
Rat anti-Embryonic lethal abnormal vision (ELAV): This antibody was developed by O'Neill et al., 1994 (Cell) and is distributed by DSHB. https://dshb.biology.uiowa.edu/Rat-Elav-7E8A10-anti-elav
Mouse anti-Reversed polarity (REPO): This antibody was developed by Alfonso and Jones 2002 (Dev. Biol.) and is distributed by DSHB. https://dshb.biology.uiowa.edu/8D12-anti-Repo
Rabbit anti-Myoinhibitory Peptide (MIP): This antibody was developed and first used by Predel et al., 2001 (Peptides).

Goat anti-rabbit AlexaFluor 488: https://www.thermofisher.com/antibody/product/Goat-anti-Rabbit-IgG-H-L-Cross-Adsorbed-Secondary-Antibody-Polyclonal/A-11008
Donkey anti-chicken AlexaFluor 488: https://www.jacksonimmuno.com/catalog/products/703-545-155
Donkey anti-rabbit AlexaFluor 546: https://www.thermofisher.com/antibody/product/Donkey-anti-Rabbit-IgG-H-L-Highly-Cross-Adsorbed-Secondary-Antibody-Polyclonal/A10040
Goat anti-mouse AlexaFluor 546: https://www.thermofisher.com/antibody/product/Goat-anti-Mouse-IgG-H-L-Highly-Cross-Adsorbed-Secondary-Antibody-Polyclonal/A-11030
Goat anti-rabbit AlexaFluor 633: https://www.thermofisher.com/antibody/product/Goat-anti-Rabbit-IgG-H-L-Cross-Adsorbed-Secondary-Antibody-Polyclonal/A-21070
Goat anti-mouse AlexaFluor 633: https://www.thermofisher.com/antibody/product/Goat-anti-Mouse-IgG-H-L-Cross-Adsorbed-Secondary-Antibody-Polyclonal/A-21050
Donkey anti-rat AlexaFluor 647:https://www.abcam.com/donkey-rat-igg-hl-alexa-fluor-647-preadsorbed-ab150155.html

## Animals and other research organisms

Policy information about studies involving animals; ARRIVE guidelines recommended for reporting animal research, and Sex and Gender in Research

Laboratory animals

Only Drosophila melanogaster strains were used for this study. All adult animals tested were between 1-5 days old; the only exception to this were the larval animals tested in Supplementary Figure 7c, which were ~72hours after fertilization. All relevant information pertaining to the parental D. melanogaster stocks (including genotypes, origins/citations, and relevant identifiers) are provided in Table 1. A complete table of each animal's genotype used for each experiment is included in Supplementary Table 1.

Wild animals

No wild animals were used in this study.

Reporting on sex

Equal numbers of male and female animals were used when possible. However, due to genetic limitations, only females were used for clonal analysis. Similarly, only females (which are larger and more amenable for in vivo physiology) were used for live-imaging experiments. Sex was considered when performing mating status comparisons, wherein: (1) "virgin females" denotes females that were meconium-positive upon collection, (2) non-virgin females were housed with males until processing for immunohistochemistry, and (3) flies were age-matched and kept on similar media until being processed for immunohistochemistry.

Sex based analyses included in this study include: (1) SPR-GAL4::VP16 expression in antennae and maxillary palps significantly differs between males, mated females, and virgin females; (2) SPR-GAL4::VP16 expression in glutamatergic LNs between males, mated females, and virgin females; (3) SPR-T2A-GAL4 expression in maxillary palps significantly differs between males, mated females, and virgin females; (4) the number of MIPergic LNs differ between males, mated females, and virgin females; and, (5) SPR-T2A-GAL4 expression in antennae between males, mated females, and virgin females.

Data disaggregated for sex is provided in the main manuscript where possible.

Field-collected samples

No samples collected from the field were used in this study.

Ethics oversight

Use of invertebrate D. melanogaster did not require ethical approval or guidance.

Note that full information on the approval of the study protocol must also be provided in the manuscript.
